# Impact of the COVID-19 pandemic on the lifestyle, mental health, and quality of life of adults in South Korea

**DOI:** 10.1371/journal.pone.0247970

**Published:** 2021-02-26

**Authors:** Kang-Hyun Park, Ah-Ram Kim, Min-Ah Yang, Seung-Ju Lim, Ji-Hyuk Park

**Affiliations:** Department of Occupational Therapy, Yonsei University, Wonju, South Korea; National Cheng Kung University College of Medicine, TAIWAN

## Abstract

**Objective:**

The COVID-19 pandemic continues to pose significant challenges to nations. The Korean government aimed to mitigate the spread of COVID-19 through stay-at-home strategies and maintaining social distance, which are likely to result in major changes in the lifestyle, mental health, and quality of life of citizens. This study aimed to investigate the impact of the COVID-19 pandemic on these factors in Koreans over 20 years old.

**Methods:**

The study sample consisted of 104 adults in South Korea aged over 20 years. An online survey was conducted between August and October 2020, in which participants were asked to complete the Yonsei Lifestyle Profile to assess lifestyle changes, the Center for Epidemiological Studies-Depression Scale, and the World Health Organization Quality of Life Scale abbreviated version. To investigate the changes in people’s lifestyles, depression, and quality of life post COVID-19, descriptive statistics were calculated for these indicators before and after the onset of the pandemic. The p-value was two-sided, and values <0.05, were regarded as statistically significant.

**Results:**

There was a significant decline in physical and other meaningful activities, including activities of daily living, leisure, social activity, and education. However, there were no significant changes in nutrition, except in the consumption of carbohydrates and minerals. Participants reported that their quality of life and mental health had decreased after the pandemic struck.

**Conclusions:**

We obtained novel data on the changes in the lifestyle, mental health, and quality of life of South Korean adults before and after the onset of the pandemic. The results of our study may assist health policymakers and practitioners in the development of health education or relevant interventions to deal with the pandemic situation as well as future crises.

## Introduction

COVID-19 originated in Wuhan, China, and as the prevalence of human-to-human propagation intensified, the World Health Organization (WHO) declared a pandemic on March 11, 2020 [1]. The disease affects all ages [2, 3], and countries have issued policies to prevent infections, such as “social distancing” and “staying at home” [4]. Previous studies have demonstrated the necessity of preventing the spread of COVID-19 in areas with high population densities through different control measures [5–7]. For this reason, South Korea has applied various regulations such as social distancing, working from home, and administrative orders to limit gatherings in order to avoid high population densities [8].

COVID-19 leads to isolation because people have to remain at home to prevent infection, but this is likely to have a detrimental effect on the physical and mental health of individuals [9]. In particular, previous research has demonstrated government actions related to spatial distancing as being effective public health measures; however, they could also cause health problems other than COVID-19 infection such as psychological distress and fear [10]. Health must be considered in these circumstances since there is no reliable cure for this disease yet, and apart from vaccination, its resolution remains unpredictable. Therefore, it is essential to prioritize the preventive approach as practiced in Korea to stay protected and maintain health and wellbeing [11].

From the perspective of prevention, a healthy lifestyle is crucial [12]. Lifestyle has been defined diversely and comprehensively in research and is still being studied. According to Park (2019), lifestyle can be classified according to people’s life patterns, and can be defined as a complex concept that involves a person’s consciousness of life, values, and character [13]. Drinking, smoking, exercise, nutrition, and stress are also elements of lifestyle according to the WHO’s definition of the term [14]. Previous studies have highlighted the importance of healthy lifestyles as they are crucial in maintaining and improving physical and mental health and improving the quality of life [14, 15]. Previous research linking COVID-19 and lifestyle patterns illustrated that an individual’s lifestyle is a crucial factor for preventing infectious diseases [16].

However, most research has been conducted on lifestyle changes for certain age groups, and the number of studies on lifestyle for all age groups is insufficient [17, 18]. To prepare for future problems with infectious diseases or pandemics, it is necessary to conduct a comparative analysis before and after infectious diseases break out and take suitable measures. Additionally, among studies related to infectious diseases, there is insufficient research on the changes in lifestyles of people before and after COVID-19, and there are few studies on how infectious diseases affect lifestyles, mental health, and quality of life. Therefore, this study summarizes these factors using basic data.

The purpose of this study is to investigate the lifestyle, mental health, and quality of life of people post COVID-19. This study was conducted through online questionnaires based on lifestyle profile problems related to COVID-19, mental health, and quality of life. Furthermore, based on the study of lifestyle conditions that have changed due to the pandemic, the research aims to be used as basic data for developing countermeasures that national and local governments can take in the event of a disaster.

## Materials and methods

### Participants

This study was designed as a cross-sectional online survey. Because of the implementation of social distancing due to the COVID-19 pandemic, as well as our limited resources, we used an online survey method. The online survey was conducted using the Google Forms web survey platform. The link to the online survey questionnaire was distributed through social media platforms such as Facebook, Kakao talk, email, and the personal contacts of the research group members. We also asked participants to share the questionnaire link to increase the number of persons who received the invitation to participate in the study and therefore increase the number of participants in this study. The inclusion criteria for the study included: (1) community-dwelling adults aged over 20 years and (2) living in South Korea for the past year. Individuals who agreed to participate in the study checked the consent tick box on the first page of the survey form. Participants submitted their informed written consent via email. Those who participated were asked to complete online questionnaires and also answered questions regarding their general information and their lifestyle changes using the Yonsei Lifestyle Profile (YLP), which had been developed based on previous research [19], mental health, and quality of life. This study was conducted between August and October 2020, with a total of 104 respondents. This study was carried out with approval for ethical procedures by the Institutional Review Board of Yonsei University Mirae Campus (1041849-202008-SB-096-01).

### Measurements

This study measured multifaceted lifestyle factors among adults in South Korea using the YLP questionnaire, which had been developed in a previous study [20]. A total of 60 items of the YLP measure three different lifestyle factors: (1) physical activity, (2) participation in activities, and (3) nutrition. Respondents are asked about frequency of their participation in certain activities, and the number of times they consumed certain foods for a week. They are asked to respond to each question twice, basing their answer on their typical routines before and after the onset of COVID-19 (S1 File). In addition, satisfaction with their participation in physical activities and their participation in activities, as well as satisfaction with their consumption of nutrition were assessed. For example, respondents were asked to respond to the level of their satisfaction with their participation in physical activities, participation in other meaningful activities, and consumption of nutrition (e.g., Before the onset of the COVID-19 pandemic and after) during the last week, did you engage in aerobic exercises as you wanted?). The YLP reflected high internal reliability, with a Cronbach’s alpha of 0.83. The intraclass correlation coefficient was 0.97 for the total score of the YLP regarding test-retest reliability [20]. The full version of the questionnaire is available in S1 File.

### Lifestyle indicators

#### Physical activity

A total of six items for physical activity were assessed using a five-point Likert scale to measure the frequencies of respondents’ participation in six different physical activities and their satisfaction with their participation in these physical activities. The six physical activities included aerobic physical activity; anaerobic physical activity; low-intensity physical activity equivalent to 2–2.9 metabolic equivalent of task (MET), including gardening, house cleaning, etc.; moderate-intensity physical activity equivalent to 3–5.9 MET, including swimming, doubles tennis, etc.; high-intensity physical activity equivalent to 6–9.9MET, such as running, climbing, etc.; and walking exercises. According to the American College of Sports Medicine (ACSM), physical activity can be divided into three types, based on intensity [21]. To assess the impact of the COVID-19 pandemic on physical activity, questions were asked about the frequency and satisfaction regarding physical activity participation per week before and after the onset of COVID-19. The higher the score, the higher the level of participation and satisfaction with physical activity.

#### Activity participation

Six items for activity participation were also assessed using a five-point Likert scale to measure the frequencies and satisfaction of diversity of participation in activities, such as activities of daily living (ADLs), leisure, social activity, work, education, and sleep during the week before and after COVID-19. A higher score indicated more frequent participation in various activities, as well as higher satisfaction with participation in activities.

#### Nutrition

Finally, nine items for nutrition were assessed using a five-point Likert scale to measure nutrition during the week before and after COVID-19 in order to measure the participants’ nutritional status. The amount of carbohydrates, proteins, fats, vitamins, minerals, water, and alcohol the participants consumed, and the frequency of drinking and smoking, were measured. For example, the participants were asked, “Before the COVID-19 pandemic, how often do you consume carbohydrate-rich foods such as rice, bread and flour in the last week?” Participants answered these questions by selecting one of the choice of the five-point Likert scale: (1) never, (2) 1–2 times per week, (3) 3–4 times per week, (4) 5–6 times per week, and (5) every day. A higher score indicated more consumption of each type of nutrition.

### Mental health indicator

Depression, the most commonly used mental health indicator, was measured using the Center for Epidemiological Studies-Depression Scale (CES-D) [22], which is one of the most widely used self-reporting tools for evaluating depression in the general population [22, 23]. It consists of 20 items related to depressive symptoms. Each item is rated on a four-point scale, ranging from 0 to 3, wherein 0, 1, 2, and 3 stand for “*rarely or none of the time*,” “*some or a little of the time*,” “*occasionally or a moderate amount of time*,” and “*most or all of the time*,” respectively. The total possible score ranges from 0to 60, with higher scores indicating more symptoms of depression by frequency of occurrence in the past week. The Cronbach’s alphas were 0.91 and 0.94 before and after the onset COVID-19, respectively.

### Quality of life indicator

The World Health Organization quality of life scale abbreviated version (WHOQOL-BREF) was used to evaluate quality of life [24, 25]. It contains 26 items rated on a five-point Likert scale and measures four domains: physical, psychological, social, and environmental. Raw domain scores were converted to a scale ranging from 0 to 100 to facilitate comparison with other instruments, with higher scores indicating a higher quality of life. The Cronbach’s alphas were 0.91 and 0.94 before and after COVID-19, respectively.

### Data analysis

To investigate changes in lifestyle, depression, and quality of life due to COVID-19, this study calculated the descriptive statistics of indicators measured before and after the pandemic. A paired t-test was used for our analysis, and the confidence interval was set at 95%. The *p*-value was two-sided, and statistical significance was set at *p*<0.05. All statistical analyses were performed using SPSS software version 25.0 (SPSS Institute, Cary, NC, USA). Missing data was processed using pairwise deletions. Therefore, the remaining items were used in the analysis, expect for the data of those who did not respond.

## Results

### Characteristics of the study population

Of the study participants, 75 (72.12%) were women. As for age groups, individuals aged 20–29, 30–39, 40–49, and over 50 made up 45.19%, 44.23%, 5.77%, and 4.81% of the sample, respectively. Most of the respondents were educated beyond college (92.31%), and half of the respondents lived in metropolitan areas (50.00%). Twenty respondents were taking medication regularly (19.23%) (Table 1).

**Table 1:** General characteristics (N = 104).

Variables		n	(%)
Sex	Male	29	27.88
	Female	75	72.12
Age	20–29	47	45.19
	30–39	46	44.23
	40–49	6	5.77
	Over 50	5	4.81
	Mean(SD)	32.04(7.64)	
Educational level	High school	8	7.69
	College	96	92.31
Residential area	Metropolitan	52	50
	Provincial big city	27	25.96
	Small and medium-sized cities	20	19.23
	Countryside	5	4.81
Medication-taking	No	84	80.77
	Yes	20	19.23

### Change in physical activity due to COVID-19

Physical activity was measured using a questionnaire constructed for this study (Table 2). There was a significant decrease in the level of aerobic, anaerobic, low-intensity, high-intensity, and walking exercises for all aspects of performance, including days performed, time, and satisfaction (*p*<0.001). The time and number of days when low-intensity exercise was performed (*p*<0.01), as well as satisfaction with exercise (*p*<0.00), decreased significantly. These results confirmed a significant decrease in physical activity due to COVID-19.

**Table 2:** Change in physical activity due to COVID-19.

		Before		After		*t* (95% Confidence interval)	*p*-value
		*M*	*SD*	*M*	*SD*		
Aerobic exercise	1	2.48	0.95	1.88	0.87	6.37 (0.42–0.80)	p<0.001
	2	3.07	1.29	2.23	1.25	6.41 (0.58–1.09)	p<0.001
	3	2.65	0.82	1.90	0.89	6.93 (0.53–0.96)	p<0.001
Anaerobic exercise	1	1.97	1.02	1.68	0.98	3.12 (0.10–0.47)	0.002
	2	2.35	1.37	1.84	1.17	4.17 (0.27–0.75)	p<0.001
	3	2.50	0.95	1.97	1.00	4.20 (0.28–0.78)	p<0.001
Low-intensity exercise	1	2.88	1.11	2.58	1.20	3.59 (0.13–0.46)	p<0.001
	2	2.87	1.03	2.62	1.11	2.65 (0.06–0.44)	0.009
	3	2.98	0.70	2.59	1.07	3.84 (0.20–0.60)	p<0.001
Moderate-intensity exercise	1	1.73	0.96	1.30	0.07	5.45 (0.27–0.60)	p<0.001
	2	2.13	1.45	1.30	0.77	6.20 (0.56–1.09)	p<0.001
	3	2.30	1.05	1.63	0.86	6.63 (0.46–0.86)	p<0.001
High-intensity exercise	1	1.73	0.91	1.40	0.77	4.49 (0.18–0.47)	p<0.001
	2	2.16	1.41	1.58	1.14	4.12 (0.30–0.87)	p<0.001
	3	2.48	0.99	1.86	0.91	5.90 (0.41–0.83)	p<0.001
Walking exercise	1	2.82	1.16	2.22	0.94	5.31 (0.37–0.82)	p<0.001
	2	2.92	1.07	2.33	1.07	5.47 (0.38–0.81)	p<0.001
	3	2.89	0.80	2.09	0.89	6.61 (0.56–1.05)	p<0.001

1 = Days performed, 2 = Performance time, 3 = Performance satisfaction.

### Change in participation due to COVID-19

Social participation was measured using a questionnaire constructed for this study (Table 3). The number of days and time performing ADLs and leisure, social, and educational activities decreased significantly (*p*<0.001). As for the number of days and time when work was performed, no significant change was observed (*p*>0.05); however, satisfaction increased significantly (*p*<0.001). Finally, there was no significant change in the number of days of sleep performance (*p*>0.05), while performance time decreased significantly (*p*<0.01).

**Table 3:** Change in participation due to COVID-19.

		Before		After		*t* (95% Confidence interval)	*p*-value
		*M*	*SD*	*M*	*SD*		
ADL	1	4.26	0.88	3.72	1.22	5.09 (0.33–0.75)	p<0.001
	2	4.64	0.70	4.27	1.07	4.04 (0.20–0.56)	p<0.001
	3	2.65	0.82	3.44	1.19	-6.46 (-1.03 − -0.55)	p<0.001
Leisure	1	2.44	0.83	1.63	0.85	10.45 (0.66–0.97)	p<0.001
	2	4.15	1.17	2.38	1.61	10.36 (1.43–2.11)	p<0.001
	3	3.02	0.81	4.40	0.95	-12.84 (-1.56 − -1.17)	p<0.001
Social activity	1	2.53	0.95	1.57	0.80	13.32 (0.82–1.10)	p<0.001
	2	4.20	1.23	2.36	1.58	11.95 (1.54–2.15)	p<0.001
	3	2.79	0.88	4.29	1.01	-12.43 (-1.74 − -1.26)	p<0.001
Work	1	3.40	1.19	3.20	1.24	2.60 (0.05–0.35)	0.011
	2	4.30	1.52	4.17	1.59	1.25 (-0.07–0.32)	0.215
	3	2.50	1.20	2.90	1.43	-3.97 (-0.61 − -0.20)	p<0.001
Education	1	2.29	0.94	1.95	1.14	3.77 (0.16–0.51)	p<0.001
	2	3.75	1.61	2.83	1.86	5.22 (0.57–1.27)	p<0.001
	3	3.11	1.00	3.84	1.08	-5.60 (-0.99–0.47)	p<0.001
Sleep	1	2.65	0.66	2.81	0.81	-2.31 (-0.27 − -0.02)	0.023
	2	3.41	0.81	3.14	1.07	2.96 (0.09–0.45)	0.004

1 = Days performed, 2 = Performance time, 3 = Performance satisfaction.

### Change in food intake due to COVID-19

Food intake was measured using the questionnaires constructed for this study (Table 4). Among the nutrients included in this study, the intake of carbohydrates and minerals increased significantly (*p*<0.05), while no significant differences were observed in the consumption of protein, fat, and vitamins (*p*>0.05). There were also no significant differences in water intake, smoking, and drinking (*p*>0.05). The amount of alcohol consumed significantly decreased after the onset of the COVID-19 pandemic (*p*<0.05).

**Table 4:** Change in food intake according to COVID-19.

	Before		After		*t* (95% Confidence interval)	*p*-value
	*M*	*SD*	*M*	*SD*		
Carbohydrate	2.93	0.50	2.99	.055	-2.10 (-0.01 − -0.00)	0.038
Protein	2.49	0.53	2.48	0.56	0.21 (-0.05–0.06)	0.830
Fat	1.98	0.56	1.99	0.57	-0.30 (-0.05–0.03)	0.765
Vitamin	2.66	0.75	2.73	0.69	-1.60(-0.16–0.02)	0.113
Minerals	2.07	0.57	2.15	0.58	-2.50(-0.16–0.02)	0.014
Water (8 cups)	3.01	1.39	3.14	1.33	-1.53(-0.30–0.04)	0.129
Smoking	1.46	1.24	1.47	1.24	-1.00(-0.03–0.24)	0.320
Drinking	1.73	0.77	1.62	0.81	1.58(-0.03–0.24)	0.117
Alcohol	2.64	1.42	2.42	1.41	2.63(0.05–0.39)	0.010

### Change in quality of life due to COVID-19

**Fig 1:**
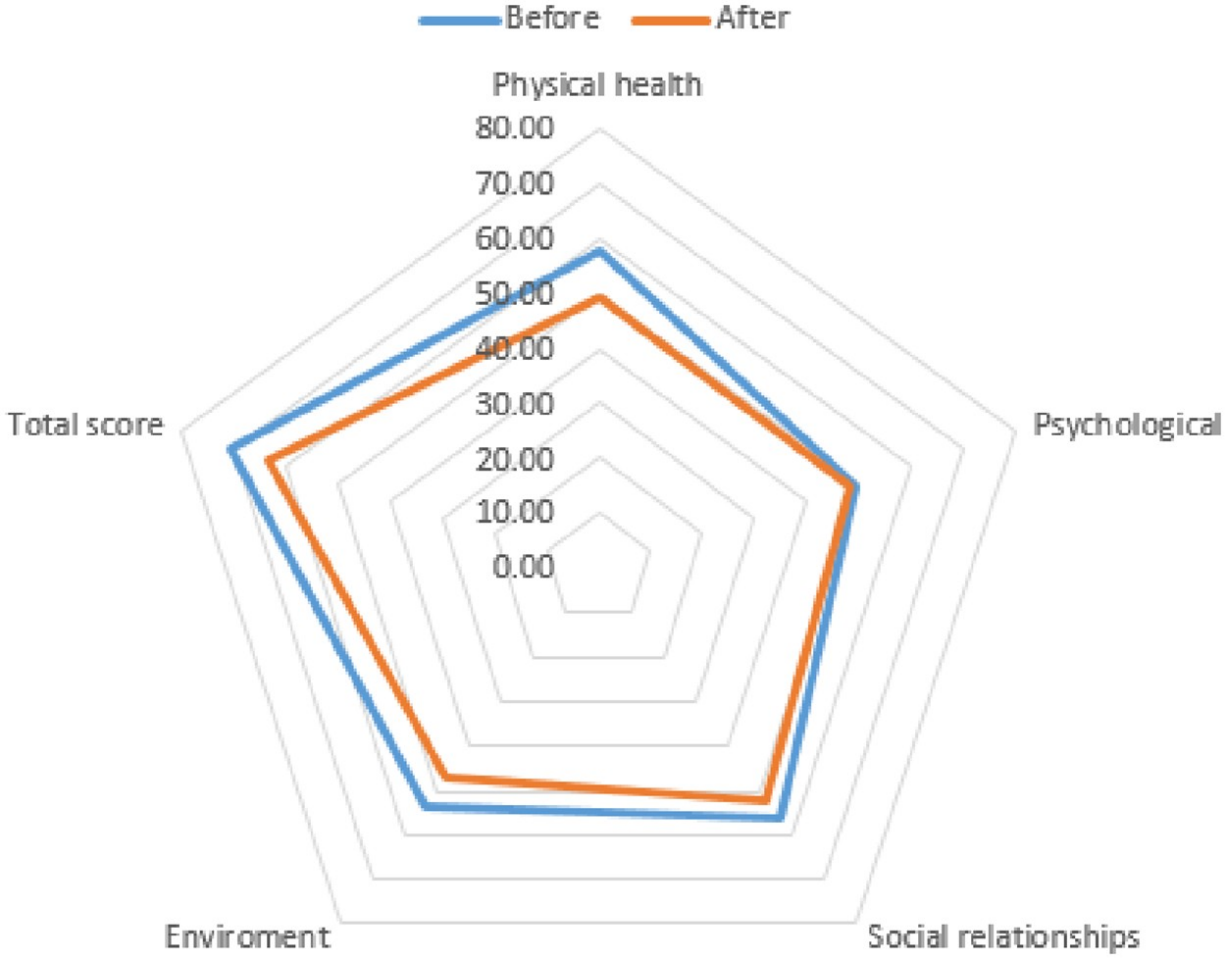
Change in WHOQOL-BREF scores according to COVID-19.

The quality of life of the study participants was measured using the WHOQOL-BREF (Table 5). The mean of each item and total score decreased during the pandemic (Fig 1). Comparing the results before and after COVID-19, there were significant decreases in the following sub-items: physical health, social relationships, environment, and general (*p*<0.001). The psychological quality of life decreased, but this was not statistically significant (*p*>0.05). The WHOQOL-BREF total scores showed a significant decrease after the outbreak of COVID-19 (*p*<0.001). These results indicate a decrease in the quality of life due to the pandemic situation.

**Table 5:** Change in quality of life due to COVID-19.

	Before		After		*t (95% Confidence interval)*	*p*-value
	*M*	*SD*	*M*	*SD*		
Physical health	20.15	3.35	17.29	3.94	8.47 (2.19–3.53)	p<0.001
Psychological	14.70	2.57	14.46	3.74	0.87 (-0.30–0.77)	0.386
Social relationships	8.42	1.31	7.82	1.68	4.54 (0.33–0.86)	p<0.001
Environment	21.46	4.37	18.76	4.62	7.06 (1.95–3.47)	p<0.001
General	5.41	1.25	4.68	1.42	5.90 (0.49–0.98)	p<0.001
Total score	70.15	10.92	63.00	13.10	7.18 (5.17–9.12)	p<0.001

### Change in depression due to COVID-19

**Fig 2:**
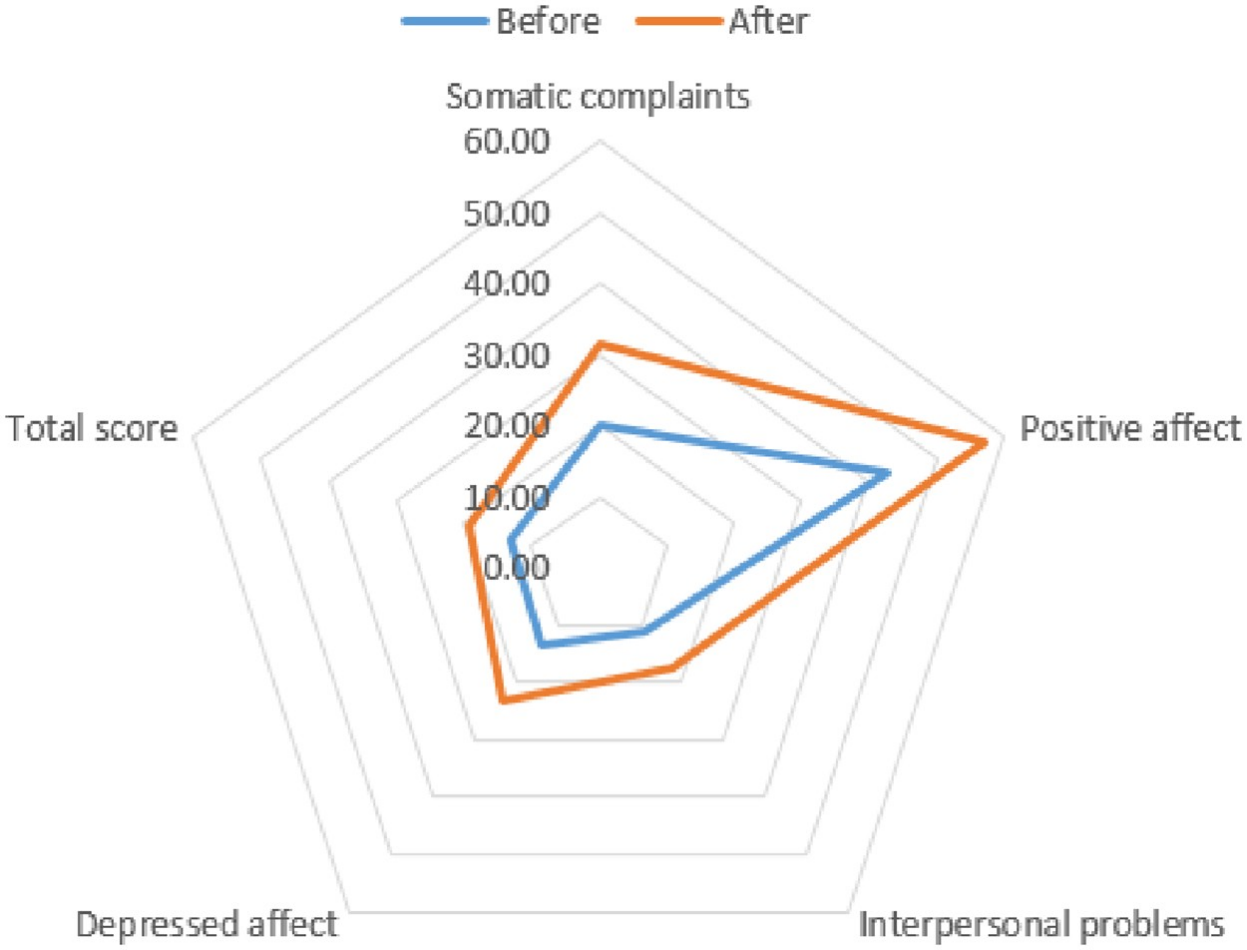
Change in CES-D10 scores according to COVID-19.

Depression among the study participants was measured using the CES-D (Table 6). Comparing the mean of each item and the total score on the CES-D, the mean and total points of each item during COVID-19 decreased (Fig 2). When compared before and after COVID-19, the depression scores of the participants significantly increased in all sub-categories (*p*<0.001). In addition, significant changes were observed when total scores were compared (*p*<0.001). This indicates that the higher the total score, the greater the severity of depression, and the more likely that depression was caused by the pandemic situation.

**Table 6:** Change in depression due to COVID-19.

	Before		After		*t* (95% Confidence interval)	*p*-value
	*M*	*SD*	*M*	*SD*		
Somatic complaints	4.85	4.12	7.59	5.23	-5.74 (-3.69 − -1.80)	p<0.001
Positive affect	5.13	2.95	6.88	3.13	-7.10 (-2.24 − -1.26)	p<0.001
Interpersonal problems	1.35	1.75	2.13	2.21	-4.69 (-1.11 − -0.45)	p<0.001
Depressed affect	1.62	1.97	2.78	2.40	-5.73 (-1.57 − -0.76)	p<0.001
Total score	12.93	9.04	19.37	11.34	-6.67 (-8.35 − -4.52)	p<0.001

## Discussion

This study provides a timely investigation of changes in the multifaceted lifestyles (physical activity, participation in activities, and nutrition) of adults during the COVID-19 pandemic in South Korea. The major findings of this study indicate that COVID-19 has had a negative impact on healthy and active lifestyles, as well as mental health and quality of life. This study illustrated significant reductions in physical activities and activity participation, such as activities of daily living, social activity, leisure, and education. Conversely, eating habits (nutrition) did not significantly changed. However, mental health and quality of life also decreased according to individual lifestyles during the pandemic.

This study showed that the frequency and time of all types of physical activities decreased during the pandemic compared to earlier periods. Unsurprisingly, satisfaction with participation in physical activities decreased. The findings of our study regarding reduced physical activity during the pandemic follow those of other studies [26–29]. Similarly, Gornicka et al. reported that COVID-19 had a negative effect on physical activity in adults. These results are consistent with a previous study in Canada that reported a significant reduction in all physical activities in children and adolescents [29]. Various restrictions to prevent the spread of COVID-19, including home confinement and social distancing, worked to reduce the overall physical activity level. It has been established that reduced physical activity leads to increased body weight and risk of illness, including inflammatory and cardiometabolic diseases [30, 31]. Several studies have demonstrated that patients with metabolic disorders have a higher risk of contracting the disease [32, 33].

The pandemic has also brought about significant changes in daily living patterns among adults in South Korea. With changed daily schedules caused by social distancing, the closure of colleges, universities, and shops, and telecommuting, participants revealed changes in how they preoccupied themselves, in which they tended to spend less time on social activities, leisure, and education. The total time participants spent sleeping was significantly higher than that before the pandemic. However, although their total sleep time increased, satisfaction with sleep decreased. This implies that the participants may have had poor sleep quality or patterns. According to an Australian report, 40.7% of Australian adults reported a negative change in sleep patterns since the onset of the pandemic [34]. The imbalance in occupation and changed sleep routines can have negative effects on individuals’ health and quality of life [35–37]; thus, thus it is necessary to provide appropriate strategies to rebuild balanced lifestyle patterns.

Eating habits also changed during the pandemic; in particular, participants consumed significantly more carbohydrates and minerals and significantly less alcohol. However, there were no significant changes in the consumption of protein, fat, vitamins, and water. According to previous research [26], this phenomenon can be explained by social distancing and other restrictions, giving individuals more opportunities to prepare meals at home. In modern society, people lack the time to prepare meals because of their busy lifestyles [38].

Our study illustrated that different factors that affect individuals’ lifestyle including physical activity, participation in activities, and nutrition, changed significantly before and after the pandemic, which led participants to lead not only a sedentary life but also an imbalanced one. According to a previous study, this changed lifestyle pattern had a negative effect on mental health and quality of life [39]. In particular, Lau et al. reported that there was a negative impact on the mental health and quality of life in residents living in Hong Kong during the severe acute respiratory syndrome (SARS) epidemic in 2003 [39]. The participants of the current study also demonstrated that there was a change in depression status before and after the pandemic; they reported increased depressive symptoms during this period. Moreover, participants’ quality of life was reduced. In future studies, the correlation between the changed lifestyle caused by the COVID-19 pandemic and mental health and lifestyle should be analyzed.

Our findings should be considered in the context of their strengths and limitations. One strength of this study is its novelty; to the best of the researchers’ knowledge, this is the first study on the multifaceted lifestyle changes that occurred before and after the pandemic in South Korea. We used an online survey, which was an ideal research tool that allowed the recruitment of samples from different regions in South Korea without increasing the risk of coronavirus transmission. However, this study had several limitations. First, the small sample size limits to generalizability of our findings to the entire South Korean population. In addition, participants in our sample were younger, healthy, and had a higher education level than the general population. This is partly because our sample was a convenience sample limited to those with social media accounts such as Facebook, Kakao Talk, or email addresses. Because of the study method, only people with Internet access could participate in this study; thus participation was not limited for people without Internet access. Hence, the demographics of our sample may not completely represent the South Korean general public. Consequently, our findings should be interpreted cautiously, and their generalization to a wider population may be limited. Second, subjective measures were used to assess lifestyle. Although all the questionnaires used in this study had been previously validated, other objective measures should be included in future research to assess lifestyle patterns accurately. Finally, this study was limited to measure the participants’ views about COVID-19. Previous research has reported that the different perspective of different people regarding COVID-19 might impact their daily lifestyles, mental health, and quality of life [40, 41]. Therefore, in future studies, peoples’ perception of COVID-19 should be considered by conducting relevant assessments, using tools such as the Fear of the COVID-19 Scale (FCoV-19S) [10].

## Conclusion

In this study, we provided novel data on patterns in the changes in the lifestyle of the South Korean population before and after the onset of the COVID-19 pandemic. Decreased participation in physical activities and meaningful activities such as social activities, leisure, and education, were identified among South Korean adults during this period. Given that the pandemic is still ongoing, these findings may have crucial public health implications and provide evidence for the development of future intervention studies.

## Supporting information

S1 File (DOCX)
