# Supplementary material for: Impact of the COVID-19 pandemic on the lifestyle, mental health, and quality of life of adults in South Korea
Source: PLoS One. 2021 Feb 26;16(2):e0247970. doi: 10.1371/journal.pone.0247970 (PMC7909697; doi:10.1371/journal.pone.0247970)
Supplement: S1 File — (DOCX) [file pone.0247970.s001.docx]

S1 File. The Yonsei Lifestyle Profile (English version)

**The Yonsei Lifestyle Profile**

**Ⅰ. Physical activity**

The following are questions about physical activity. Indicate how many times you participate in the following activities per week.

Please consider your engagement in these activities before and after the onset of the COVID-19 pandemic and mark (∨) your answer on the box next to the choice that is most applicable for you.

**1. This is a question about aerobic exercise in the last week.**

(※ Aerobic exercise: A relatively long, continuous breathing exercise, such as jogging, aerobics, jump rope, etc.)

1.1) (Before COVID-19) How many days did you engage in aerobic exercises in the last week?

| **□ Never** | **□ 1–2 days** | **□ 3–4 days** | **□ 5–6 days** | **□ Every day** |
| --- | --- | --- | --- | --- |

1.2) (Before COVID-19) On average, how much time did you engage in aerobic exercises during a day?

| **□ Never** | **□ 10–20 min** | **□ 21–40 min** | **□ 41–60 min** | **□ over 1 hour** |
| --- | --- | --- | --- | --- |

1.3) (Before COVID-19) Did you engage in aerobic exercises as much as you wanted?

| **□ Always less than I wanted** | **□ Sometimes less than I wanted** | **□ About right for me** | **□ Sometimes more than I wanted** | **□ Always more than I wanted** |
| --- | --- | --- | --- | --- |

1.1-1) (After COVID-19 onset) How many days did you engage in aerobic exercises in the last week?

| **□ Never** | **□ 1–2 days** | **□ 3–4 days** | **□ 5–6 days** | **□ Every day** |
| --- | --- | --- | --- | --- |

1.2-2) (After COVID-19 onset) On average, how much time did you engage in aerobic exercises during a day?

| **□ Never** | **□ 10–20 min** | **□ 21–40 min** | **□ 41–60 min** | **□ over 1 hour** |
| --- | --- | --- | --- | --- |

1.3-3) (After COVID-19 onset) Do you engage in aerobic exercises as much as you want?

| **□ Always less than I want** | **□ Sometimes less than I want** | **□ About right for me** | **□ Sometimes more than I want** | **□ Always more than I want** |
| --- | --- | --- | --- | --- |

**2. This is a question about anaerobic exercise in the last week.**

(※ Anaerobic exercise: Short-duration workouts, such as sprinting and push-ups, which leave one short of breath, and are difficult to sustain.)

2.1) (Before COVID-19) How many days did you engage in anaerobic exercises in the last week?

| **□ Never** | **□ 1–2 days** | **□ 3–4 days** | **□ 5–6 days** | **□ Every day** |
| --- | --- | --- | --- | --- |

2.2) (Before COVID-19) On average, how much time did you engage in anaerobic exercises during a day?

| **□ Never** | **□ 10–20 min** | **□ 21–40 min** | **□ 41–60 min** | **□ over 1 hour** |
| --- | --- | --- | --- | --- |

2.3) (Before COVID-19) Did you engage in anaerobic exercises as much as you wanted?

| **□ Always less than I wanted** | **□ Sometimes less than I wanted** | **□ About right for me** | **□ Sometimes more than I wanted** | **□ Always more than I wanted** |
| --- | --- | --- | --- | --- |

2.1-1) (After COVID-19 onset) How many days did you engage in anaerobic exercises in the last week?

| **□ Never** | **□ 1–2 days** | **□ 3–4 days** | **□ 5–6 days** | **□ Every day** |
| --- | --- | --- | --- | --- |

2.2-2) (After COVID-19 onset) On average, how much time did you engage in anaerobic exercises during a day?

| **□ Never** | **□ 10–20 min** | **□ 21–40 min** | **□ 41–60 min** | **□ over 1 hour** |
| --- | --- | --- | --- | --- |

2.3-3) (After COVID-19 onset) Do you engage in anaerobic exercises as much as you want?

| **□ Always less than I want** | **□ Sometimes less than I want** | **□ About right for me** | **□ Sometimes more than I want** | **□ Always more than I want** |
| --- | --- | --- | --- | --- |

**3. This is a question about low-intensity physical activity in the last week.**

(※ Low-intensity physical activity: physical activity equivalent to 2**–**2.9 MET, such as gardening, house-cleaning, fishing, etc.)

3.1) (Before COVID-19) How many days did you engage in low-intensity physical activities in the last week?

| **□ Never** | **□ 1–2 days** | **□ 3–4 days** | **□ 5–6 days** | **□ Every day** |
| --- | --- | --- | --- | --- |

3.2) (Before COVID-19) On average, how much time did you engage in low-intensity physical activities during a day?

| **□ Never** | **□ 10–20 min** | **□ 21–40 min** | **□ 41–60 min** | **□ over 1 hour** |
| --- | --- | --- | --- | --- |

3.3) (Before COVID-19) Did you engage in low-intensity physical activities as much as you wanted?

| **□ Always less than I wanted** | **□ Sometimes less than I wanted** | **□ About right for me** | **□ Sometimes more than I wanted** | **□ Always more than I wanted** |
| --- | --- | --- | --- | --- |

| **□ Never** | **□ 1–2 days** | **□ 3–4 days** | **□ 5–6 days** | **□ Every day** |
| --- | --- | --- | --- | --- |

3.1-1) (After COVID-19 onset) How many days did you engage in low-intensity physical activities in the last week?

3.2-2) (After COVID-19 onset) On average, how much time did you engage in low-intensity physical activities during a day?

| **□ Never** | **□ 10–20 min** | **□ 21–40 min** | **□ 41–60 min** | **□ over 1 hour** |
| --- | --- | --- | --- | --- |

3.3-3) (After COVID-19 onset) Do you engage in low-intensity physical activities as much as you want?

| **□ Always less than I want** | **□ Sometimes less than I want** | **□ About right for me** | **□ Sometimes more than I want** | **□ Always more than I want** |
| --- | --- | --- | --- | --- |

**4. This is a question about moderate-intensity physical activity in the last week.**

(※ Moderate-intensity physical activity: physical activity equivalent to 3–5.9 MET. such as swimming, doubles tennis, badminton, etc.)

4.1) (Before COVID-19) How many days did you engage in moderate-intensity physical activities in the last week?

| **□ Never** | **□ 1–2 days** | **□ 3****–4 days** | **□ 5–6 days** | **□ Every day** |
| --- | --- | --- | --- | --- |

4.2) (Before COVID-19) On average, how much time did you engage in moderate-intensity physical activities during a day?

| **□ Never** | **□ 10–20 min** | **□ 21–40 min** | **□ 41–60 min** | **□ over 1 hour** |
| --- | --- | --- | --- | --- |

4.3) (Before COVID-19) Did you engage in moderate-intensity physical activities as much as you wanted?

| **□ Always less than I wanted** | **□ Sometimes less than I wanted** | **□ About right for me** | **□ Sometimes more than I wanted** | **□ Always more than I wanted** |
| --- | --- | --- | --- | --- |

4.1-1) (After COVID-19 onset) How many days did you engage in moderate-intensity physical activities in the last week?

| **□ Never** | **□ 1–2 days** | **□ 3–4 days** | **□ 5–6 days** | **□ Every day** |
| --- | --- | --- | --- | --- |

4.2-2) (After COVID-19 onset) On average, how much time did you engage in moderate-intensity physical activity during a day?

| **□ Never** | **□ 10–20 min** | **□ 21–40 min** | **□ 41–60 min** | **□ over 1 hour** |
| --- | --- | --- | --- | --- |

4.3-3) (After COVID-19 onset) Do you engage in moderate-intensity physical activities as much as you want?

| **□ Always less than I want** | **□ Sometimes less than I want** | **□ About right for me** | **□ Sometimes more than I want** | **□ Always more than I want** |
| --- | --- | --- | --- | --- |

**5 This is a question about high-intensity physical activity in the last week.**

(※ High-intensity physical activity: physical activity equivalent to 6–9.9 MET, such as running, climbing, cycling, etc.)

5.1) (Before COVID-19) How many days did you engage in high-intensity physical activities in the last week?

| **□ Never** | **□ 1–2 days** | **□ 3–4 days** | **□ 5–6 days** | **□ Every day** |
| --- | --- | --- | --- | --- |

5.2) (Before COVID-19) On average, how much time did you engage in high-intensity physical activities during a day?

| **□ Never** | **□ 10–20 min** | **□ 21–40 min** | **□ 41–60 min** | **□ over 1 hour** |
| --- | --- | --- | --- | --- |

5.3) (Before COVID-19) Did you engage in high-intensity physical activities as much as you wanted?

| **□ Always less than I wanted** | **□ Sometimes less than I wanted** | **□ About right for me** | **□ Sometimes more than I wanted** | **□ Always more than I wanted** |
| --- | --- | --- | --- | --- |

5.1-1) (After COVID-19 onset) How many days did you engage in high-intensity physical activities in the last week?

| **□ Never** | **□ 1–2 days** | **□ 3–4 days** | **□ 5–6 days** | **□ Every day** |
| --- | --- | --- | --- | --- |

5.2-2) (After COVID-19 onset) On average, how much time did you engage in high-intensity physical activities during a day?

| **□ Never** | **□ 10–20 min** | **□ 21–40 min** | **□ 41–60 min** | **□ over 1 hour** |
| --- | --- | --- | --- | --- |

5.3-3) (After COVID-19 onset) Do you engage in high-intensity physical activities as much as you want?

| **□ Always less than I want** | **□ Sometimes less than I want** | **□ About right for me** | **□ Sometimes more than I want** | **□ Always more than I want** |
| --- | --- | --- | --- | --- |

**6. This is a question about going on walks in the last week.**

6.1) (Before COVID-19) How many days did you spend walking in the last week?

| **□ Never** | **□ 1–2 days** | **□ 3–4 days** | **□ 5–6 days** | **□ Every day** |
| --- | --- | --- | --- | --- |

6.2) (Before COVID-19) On average, how much time did you spend walking during a day?

| **□ Never** | **□ 10–20 min** | **□ 21–40 min** | **□ 41–60 min** | **□ over 1 hour** |
| --- | --- | --- | --- | --- |

6.3) (Before COVID-19) Did you walk as much as you wanted?

| **□ Always less than I wanted** | **□ Sometimes less than I wanted** | **□ About right for me** | **□ Sometimes more than I wanted** | **□ Always more than I wanted** |
| --- | --- | --- | --- | --- |

6.1-1) (After COVID-19 onset) How many days did you spend walking in the last week?

| **□ Never** | **□ 1–2 days** | **□ 3–4 days** | **□ 5–6 days** | **□ Every day** |
| --- | --- | --- | --- | --- |

6.2-2) (After COVID-19 onset) On average, how much time did you spend on walking during a day?

| **□ Never** | **□ 10–20 min** | **□ 21–40 min** | **□ 41–60 min** | **□ over 1 hour** |
| --- | --- | --- | --- | --- |

6.3-3) (After COVID-19 onset) Do you walk as often as you want?

| **□ Always less than I want** | **□ Sometimes less than I want** | **□ About right for me** | **□ Sometimes more than I want** | **□ Always more than I want** |
| --- | --- | --- | --- | --- |

**Ⅱ. Activity participation**

The following are questions about your participation in certain activities. Indicate how often you have participated in the following physical activities in the last week.

Please consider your engagement in these activities before and after the onset of the COVID-19 pandemic, and mark (∨) your answer on the box next to the choice that is most applicable for you.

**1. This is a question about activities of daily living (ADLs) in the last week.**

1.1) (Before COVID-19) How many days did you participate in ADLs in the last week?

| **□ Never** | **□ 1–2 days** | **□ 3–4 days** | **□ 5–6 days** | **□ Every day** |
| --- | --- | --- | --- | --- |

1.2) (Before COVID-19) On average, how much time did you spend on ADLs during a day?

| **□ Never** | **□ 10–20 min** | **□ 21–40 min** | **□ 41–60 min** | **□ over 1 hour** |
| --- | --- | --- | --- | --- |

1.3) (Before COVID-19) Did you participate in ADLs as much as you want?

| **□ Always less than I wanted** | **□ Sometimes less than I wanted** | **□ About right for me** | **□ Sometimes more than I wanted** | **□ Always more than I wanted** |
| --- | --- | --- | --- | --- |

1.1-1) (After COVID-19 onset) How many days did you participate in ADLs in the last week?

| **□ Never** | **□ 1–2 days** | **□ 3–4 days** | **□ 5–6 days** | **□ Every day** |
| --- | --- | --- | --- | --- |

1.2-2) (After COVID-19 onset) On average, how much time did you spend on ADLs during a day?

| **□ Never** | **□ 10–20 min** | **□ 21–40 min** | **□ 41–60 min** | **□ over 1 hour** |
| --- | --- | --- | --- | --- |

1.3-3) (After COVID-19 onset) Do you participate in ADLs as much as you want?

| **□ Always less than I want** | **□ Sometimes less than I want** | **□ About right for me** | **□ Sometimes more than I want** | **□ Always more than I want** |
| --- | --- | --- | --- | --- |

**2. This is a question about leisure activity in the last week.**

2.1) (Before COVID-19) How many days did you participate in leisure activities in the last week?

| **□ Never** | **□ 1–2 days** | **□ 3–4 days** | **□ 5–6 days** | **□ Every day** |
| --- | --- | --- | --- | --- |

2.2) (Before COVID-19) On average, how much time did you spend on leisure activities during a day?

| **□ Never** | **□ 10–20 min** | **□ 21–40 min** | **□ 41–60 min** | **□ over 1 hour** |
| --- | --- | --- | --- | --- |

2.3) (Before COVID-19) Did you participate in leisure activities as much as you wanted?

| **□ Always less than I wanted** | **□ Sometimes less than I wanted** | **□ About right for me** | **□ Sometimes more than I wanted** | **□ Always more than I wanted** |
| --- | --- | --- | --- | --- |

2.1-1) (After COVID-19 onset) How many days did you participate in leisure activities in the last week?

| **□ Never** | **□ 1–2 days** | **□ 3–4 days** | **□ 5–6 days** | **□ Every day** |
| --- | --- | --- | --- | --- |

2.2-2) (After COVID-19 onset) On average, how much time did you spend on leisure activities during a day?

| **□ Never** | **□ 10–20 min** | **□ 21–40 min** | **□ 41–60 min** | **□ over 1 hour** |
| --- | --- | --- | --- | --- |

2.3-3) (After COVID-19 onset) Do you participate in leisure activities as much as you want?

| **□ Always less than I want** | **□ Sometimes less than I want** | **□ About right for me** | **□ Sometimes more than I want** | **□ Always more than I want** |
| --- | --- | --- | --- | --- |

**3. This is a question about social activity in the last week.**

3.1) (Before COVID-19) How many days did you participate in social activities in the last week?

| **□ Never** | **□ 1–2 days** | **□ 3–4 days** | **□ 5–6 days** | **□ Every day** |
| --- | --- | --- | --- | --- |

3.2) (Before COVID-19) On average, how much time did you spend on social activities during a day?

| **□ Never** | **□ 10–20 min** | **□ 21–40 min** | **□ 41–60 min** | **□ over 1 hour** |
| --- | --- | --- | --- | --- |

3.3) (Before COVID-19) Did you participate in social activities as much as you wanted?

| **□ Always less than I wanted** | **□ Sometimes less than I wanted** | **□ About right for me** | **□ Sometimes more than I wanted** | **□ Always more than I wanted** |
| --- | --- | --- | --- | --- |

3.1-1) (After COVID-19 onset) How many days did you participate in social activities in the last week?

| **□ Never** | **□ 1–2 days** | **□ 3–4 days** | **□ 5–6 days** | **□ Every day** |
| --- | --- | --- | --- | --- |

3.2-2) (After COVID-19 onset) On average, how much time did you spend on social activities during a day?

| **□ Never** | **□ 10–20 min** | **□ 21–40 min** | **□ 41–60 min** | **□ over 1 hour** |
| --- | --- | --- | --- | --- |

3.3-3) (After COVID-19 onset) Do you participate in social activities as much as you want?

| **□ Always less than I want** | **□ Sometimes less than I want** | **□ About right for me** | **□ Sometimes more than I want** | **□ Always more than I want** |
| --- | --- | --- | --- | --- |

**4. This is a question about productive activity in the last week.**

4.1) (Before COVID-19) How many days did you participate in productive activities in the last week?

| **□ Never** | **□ 1–2 days** | **□ 3–4 days** | **□ 5–6 days** | **□ Every day** |
| --- | --- | --- | --- | --- |

4.2) (Before COVID-19) On average, how much time did you spend on productive activities during a day?

| **□ Never** | **□ 10–20 min** | **□ 21–40 min** | **□ 41–60 min** | **□ over 1 hour** |
| --- | --- | --- | --- | --- |

4.3) (Before COVID-19) Did you participate in productive activities as much as you wanted?

| **□ Always less than I wanted** | **□ Sometimes less than I wanted** | **□ About right for me** | **□ Sometimes more than I wanted** | **□ Always more than I wanted** |
| --- | --- | --- | --- | --- |

4.1-1) (After COVID-19 onset) How many days did you participate in productive activities in the last week?

| **□ Never** | **□ 1–2 days** | **□ 3–4 days** | **□ 5–6 days** | **□ Every day** |
| --- | --- | --- | --- | --- |

4.2-2) (After COVID-19 onset) On average, how much time did you spend on productive activities during a day?

| **□ Never** | **□ 10–20 min** | **□ 21–40 min** | **□ 41–60 min** | **□ over 1 hour** |
| --- | --- | --- | --- | --- |

4.3-3) (After COVID-19 onset) Do you participate in productive activities as much as you want?

| **□ Always less than I want** | **□ Sometimes less than I want** | **□ About right for me** | **□ Sometimes more than I want** | **□ Always more than I want** |
| --- | --- | --- | --- | --- |

**5. This is a question about education in the last week.**

5.1) (Before COVID-19) How many days did you spend engaged in educational activities in the last week?

| **□ Never** | **□ 1–2 days** | **□ 3–4 days** | **□ 5–6 days** | **□ Every day** |
| --- | --- | --- | --- | --- |

5.2) (Before COVID-19) On average, how much time did you spend on education during a day?

| **□ Never** | **□ 10–20 min** | **□ 21–40 min** | **□ 41–60 min** | **□ over 1 hour** |
| --- | --- | --- | --- | --- |

5.3) (Before COVID-19) Did you participate in education as much as you wanted?

| **□ Always less than I wanted** | **□ Sometimes less than I wanted** | **□ About right for me** | **□ Sometimes more than I wanted** | **□ Always more than I wanted** |
| --- | --- | --- | --- | --- |

5.1-1) (After COVID-19 onset) How many days did you participate in education in the last week?

| **□ Never** | **□ 1–2 days** | **□ 3–4 days** | **□ 5–6 days** | **□ Every day** |
| --- | --- | --- | --- | --- |

5.2-2) (After COVID-19 onset) On average, how much time do you spend on education during a day?

| **□ Never** | **□ 10–20 min** | **□ 21–40 min** | **□ 41–60 min** | **□ over 1 hour** |
| --- | --- | --- | --- | --- |

5.3-3) (After COVID-19 onset) Do you participate in education as much as you want?

| **□ Always less than I want** | **□ Sometimes less than I want** | **□ About right for me** | **□ Sometimes more than I want** | **□ Always more than I want** |
| --- | --- | --- | --- | --- |

**6. This is a question about sleep in the last week.**

6.1) (Before COVID-19) On average, how much time did you spend sleeping during a day?

| **□ Never** | **□ 10–20 min** | **□ 21–40 min** | **□ 41–60 min** | **□ over 1 hour** |
| --- | --- | --- | --- | --- |

6.2) (Before COVID-19) Did you sleep as much as you wanted?

| **□ Always less than I wanted** | **□ Sometimes less than I wanted** | **□ About right for me** | **□ Sometimes more than I wanted** | **□ Always more than I wanted** |
| --- | --- | --- | --- | --- |

6.1-1) (After COVID-19 onset) On average, how much time do you spend sleeping during a day?

| **□ Never** | **□ 10–20 min** | **□ 21–40 min** | **□ 41–60 min** | **□ over 1 hour** |
| --- | --- | --- | --- | --- |

6.2-2) (After COVID-19 onset) Do you sleep as much as you want?

| **□ Always less than I want** | **□ Sometimes less than I want** | **□ About right for me** | **□ Sometimes more than I want** | **□ Always more than I want** |
| --- | --- | --- | --- | --- |

**Ⅲ. Nutrition**

The following are questions about food intake. Indicate how many times a week the following types of foods have been consumed in the last week.

Please consider your food consumption before and after the onset of the COVID-19 pandemic and mark (∨) your answer on the box next to the choice that is most applicable for you.

**1. This is a question about the frequency of carbohydrate consumption in the past week.**

(※ Carbohydrate-rich food: rice, bread, flour, potato, corn, etc.)

1.1) (Before COVID-19) How often do you consume carbohydrate-rich foods in the last week?

| **□ Never** | **□ 1–2 times per week** | **□ 3–4 times per week** | **□ 5–6 times per week** | **□ Every day** |
| --- | --- | --- | --- | --- |

1.1-1) (After COVID-19 onset) How often do you consume carbohydrate-rich foods in the last week?

| **□ Never** | **□ 1–2 times per week** | **□ 3–4 times per week** | **□ 5–6 times per week** | **□ Every day** |
| --- | --- | --- | --- | --- |

**2. This is a question about the frequency of protein consumption in the last week.**

(※ Protein-rich food: chicken breasts, fish, tofu, beans, eggs, etc.)

2.1) (Before COVID-19) How often do you consume protein-rich foods in the last week?

| **□ Never** | **□ 1–2 times per week** | **□ 3–4 times per week** | **□ 5–6 times per week** | **□ Every day** |
| --- | --- | --- | --- | --- |

2.1-1) (After COVID-19 onset) How often do you consume protein-rich foods in the last week?

| **□ Never** | **□ 1–2 times per week** | **□ 3–4 times per week** | **□ 5–6 times per week** | **□ Every day** |
| --- | --- | --- | --- | --- |

**3. This is a question about the frequency of fat consumption in the last week.**

(※ Fatty food: butter, margarine, oil, nuts, etc.)

3.1) (Before COVID-19) How often do you consume fatty foods in the last week?

| **□ Never** | **□ 1–2 times per week** | **□ 3–4 times per week** | **□ 5–6 times per week** | **□ Every day** |
| --- | --- | --- | --- | --- |

3.1-1) (After COVID-19 onset) How often do you consume fatty foods in the last week?

| **□ Never** | **□ 1–2 times per week** | **□ 3–4 times per week** | **□ 5–6 times per week** | **□ Every day** |
| --- | --- | --- | --- | --- |

**4. This is a question about the frequency of vitamin consumption in the last week.**

(※ Vitamin-rich food: green vegetables, fruit, seaweed, etc.)

4.1) (Before COVID-19) How many vitamin-rich foods did you eat per week?

| **□ Never** | **□ 1–2 times per week** | **□ 3–4 times per week** | **□ 5–6 times per week** | **□ Every day** |
| --- | --- | --- | --- | --- |

4.1-1) (After COVID-19 onset) How many vitamin-rich foods you eat per week?

| **□ Never** | **□ 1–2 times per week** | **□ 3–4 times per week** | **□ 5–6 times per week** | **□ Every day** |
| --- | --- | --- | --- | --- |

**5. This is a question about the frequency of mineral consumption in the last week.**

(※ Mineral-rich food: dairy products, anchovies, cheese, etc.)

5.1) (Before COVID-19) How often do you consume mineral-rich food in the last week?

| **□ Never** | **□ 1–2 times per week** | **□ 3–4 times per week** | **□ 5–6 times per week** | **□ Every day** |
| --- | --- | --- | --- | --- |

5.1-1) (After COVID-19 onset) How often do you consume mineral-rich food in the last week?

| **□ Never** | **□ 1–2 times per week** | **□ 3–4 times per week** | **□ 5–6 times per week** | **□ Every day** |
| --- | --- | --- | --- | --- |

**6. This is a question about the amount of water you have consumed in the last week.**

6.1) (Before COVID-19) How many glasses of water did you drink per day?

| **□ Never** | **□ 1–3 glasses** | **□ 4–6 glasses** | **□ 7–9 glasses** | **□ over 10 glasses** |
| --- | --- | --- | --- | --- |

6.1-1) (After COVID-19 onset) How many glasses of water do you drink per day?

| **□ Never** | **□ 1–3 glasses** | **□ 4–6 glasses** | **□ 7–9 glasses** | **□ over 10 glasses** |
| --- | --- | --- | --- | --- |

**7. This is a question about the amount of smoking in a week.**

7.1) (Before COVID-19) How often did you smoke per week?

| **□ Never** | **□ 1–2 times** | **□ 3–4 times** | **□ 5–6 times** | **□ Every day** |
| --- | --- | --- | --- | --- |

7.1-1) (After COVID-19 onset) How often do you smoke per week?

| **□ Never** | **□ 1–2 times** | **□ 3–4 times** | **□ 5–6 times** | **□ Every day** |
| --- | --- | --- | --- | --- |

**8. This is a question about drinking habits in a week.**

8.1) (Before COVID-19) How often did you drink alcohol on average per week?

| **□ Never** | **□ 1–2 times** | **□ 3–4 times** | **□ 5–6 times** | **□ Every day** |
| --- | --- | --- | --- | --- |

8.1-1) (After COVID-19 onset) How often do you drink alcohol on average per week?

| **□ Never** | **□ 1–2 times** | **□ 3–4 times** | **□ 5–6 times** | **□ Every day** |
| --- | --- | --- | --- | --- |

**9. This is a question about the amount of alcohol you consume during a week.**

9.1) (Before COVID-19) how many glasses of water did you consume each time you drank alcohol?

| **□ Never** | **□ 1–3 glasses** | **□ 4–6 glasses** | **□ 7–9 glasses** | **□ over 10 glasses** |
| --- | --- | --- | --- | --- |

9.1) (After COVID-19 onset) how many glasses of water do you consume each time you drink alcohol?

| **□ Never** | **□ 1–3 glasses** | **□ 4–6 glasses** | **□ 7–9 glasses** | **□ over 10 glasses** |
| --- | --- | --- | --- | --- |
